# Inhibition of tumour necrosis factor alpha in the R6/2 mouse model of Huntington’s disease by etanercept treatment

**DOI:** 10.1038/s41598-019-43627-3

**Published:** 2019-05-10

**Authors:** Jeffrey Pido-Lopez, Benedict Tanudjojo, Sahar Farag, Marie-Katrin Bondulich, Ralph Andre, Sarah J. Tabrizi, Gillian P. Bates

**Affiliations:** 1 Huntington’s Disease Centre, Department of Neurodegenerative Disease and UK Dementia Research Institute, UCL Queen Square Institute of Neurology, University College London, London, WC1N 3BG UK; 2 Nuffield Department of Clinical Neurosciences, John Radcliffe Hospital, University of Oxford, Oxford, OX3 9DU UK

**Keywords:** Huntington's disease, Neuroimmunology, Immunosuppression

## Abstract

Huntington’s disease (HD) is an inherited neurodegenerative disorder caused by the expansion of the CAG repeat in exon 1 of the huntingtin (*HTT*) gene, which results in a mutant protein with an extended polyglutamine tract. Inflammation occurs in both the brain and the periphery of HD patients and mouse models, with increases in brain and/or plasma levels of neurotoxic TNFα and several other proinflammatory cytokines. TNFα promotes the generation of many of these cytokines, such as IL6, which raises the possibility that TNFα is central to the inflammatory milieu associated with HD. A number of mouse studies have reported that the suppression of chronic immune activation during HD has beneficial consequences. Here, we investigated whether TNFα contributes to the peripheral inflammation that occurs in the R6/2 mouse model, and whether the *in vivo* blockade of TNFα, via etanercept treatment, can modify disease progression. We found that etanercept treatment normalised the elevated plasma levels of some cytokines. This did not modify the progression of certain behavioural measures, but slightly ameliorated brain weight loss, possibly related to a reduction in the elevated striatal level of soluble TNFα.

## Introduction

Neurodegenerative diseases are commonly associated with inflammation in the central nervous system (CNS). This usually manifests as increased frequencies of active microglial innate immune cells and upregulated levels of various proinflammatory mediators, such as cytokines in the brain and cerebrospinal fluid (CSF)^1–7^. In the case of Huntington’s disease (HD), chronic activation of the peripheral immune system is also evident during the course of disease^1,4,8^ which could potentially exacerbate the inflammatory response seen in the CNS. In many previous studies, including our own, increases in the levels of the blood brain barrier (BBB) penetrating, neurotoxic cytokine TNFα was consistently observed in the HD brain and/or blood^1,5,8–12^. Interestingly, as well as being an autocrine, TNFα can promote the production of several cytokines, including TNFα itself^13^, along with the potentially neurotoxic interleukin (IL)6^10^ and IL1β^14^, as well as IL2 and the anti-inflammatory cytokine IL10^15^, which are also upregulated during the course of HD. These findings led to the hypothesis that the peripheral immune response contributed to the neuronal insult, with TNFα playing a protagonist role in the chronic inflammatory milieu associated with HD^1,8^.

HD is a fatal, progressive, inherited neurodegenerative disorder causing impairments in motor function, cognition and behaviour, with approximately 1 in 7,300 people affected in Western populations^16^. HD results from a CAG trinucleotide repeat expansion in exon 1 of the huntingtin (*HTT*) gene leading to an expanded polyglutamine sequence in the N-terminal region of the ubiquitously expressed HTT protein^17^. Proposed mechanisms for mutant HTT induced neuronal dysfunction and damage include excitotoxicity^18^, mitochondrial impairment^19^ and neuroinflammation^1^, amongst others. TNFα can induce neurotoxicity by impacting on these processes, providing the rationale for blocking TNFα activity as a therapeutic strategy for HD^9,12,19–21^.

TNFα is a *M*_r_ 17,000 protein that exists in cell membrane bound and soluble forms and binds to p55 and p75 TNF receptors (TNFRI and TNFRII respectively). The soluble forms of TNFR are involved in the regulation and bioavailability of TNFα while cell surface TNFR induces its biological functions^22^. Pleiotropic TNFα is important in maintaining several cellular processes such as cell proliferation, differentiation and survival, in addition to being a key regulator of the inflammatory response^23,24^. Its overproduction is frequently associated with a number of syndromes including rheumatoid arthritis and psoriasis, as well as neurodegenerative disorders such as Parkinson’s disease and Alzheimer’s disease^25–28^. TNFα is produced primarily by activated macrophages and T cells, which we have shown to be dysregulated in HD mice and/or patients^1,8,29^. Agents that target the TNFα upregulation seen in these diseases have shown promising results^30,31^ while in the case of Crohn’s disease and rheumatoid arthritis they have become the most widely used of the biological therapies currently prescribed^32,33^. Etanercept (Enbrel) is a recombinant dimer of human soluble TNFRII linked by the constant Fc portion of human immunoglobulin 1 (IgG1) and was the first anti-TNF drug to be approved for the treatment of rheumatoid arthritis by the US Food and Drug Administration. Etanercept competitively blocks the binding of TNFα as well TNFβ (lymphotoxin-α [LT-α]) to TNFR, thus inhibiting their biological activity^34^. We therefore utilised the capacity of etanercept to suppress TNFα in order to examine the specific contribution of TNFα to the chronic inflammation observed during HD in the R6/2 mouse model of the disease. We subsequently investigated the *in vivo* potential of this “off the shelf” drug as a therapeutic strategy for HD in the R6/2 mice by assessing the effect of *in vivo* drug treatment on HD pathology and disease progression.

Herein, we demonstrate that etanercept therapy effectively lowered the plasma levels of several proinflammatory cytokines that are elevated in R6/2 mice, without causing any adverse effects. Furthermore, *in vivo* treatment resulted in a deceleration of R6/2 mice brain atrophy, without impacting on motor and cognitive declines. At present, there are no disease-modifying treatments for HD, although several immunomodulatory regimens have conferred beneficial effects in improving symptoms and/or survival when used for treating HD mice^35,36^. Our results further suggest that the immune system, specifically TNFα, may contribute to HD progression, and that targeting this cytokine may slow down the brain deterioration found associated with disease. Further studies into the potential of etanercept as a therapeutic strategy for HD are required in order to determine whether this readily accessible drug might confer benefits to patients.

## Results

### Increases in plasma TNFα as well as TNFα induced inflammatory cytokines during late stage disease in R6/2 mice

**Figure 1:**
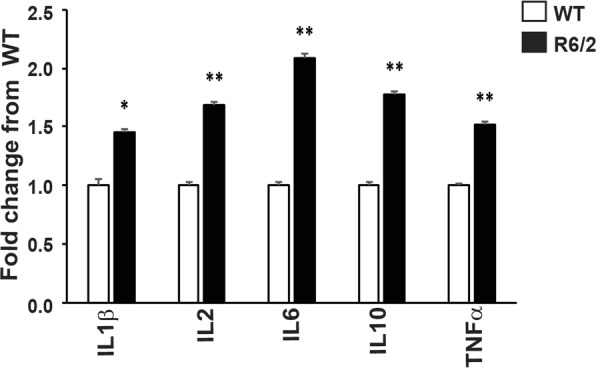
Plasma levels of TNFα and cytokines induced by TNFα are increased in late stage R6/2 mice. Increased levels of plasma TNFα, IL1β, IL2, IL6 and IL10 in late-stage R6/2 compared to WT mice at 14 weeks of age, as measured by MSD (*n* = 16–26/group). Student’s *t* test ± SEM. *p < 0.05, **p < 0.01.

We and others have shown that proinflammatory cytokine levels are increased in the blood and peripheral tissues of mouse models of HD and HD patients, indicative of chronic immune activation during the course of disease^1,8,29^. These increases occur in both R6/2 transgenic mice^1,8^, which express exon 1 HTT^37^, as well as knock-in models^1,8^ in which the mutation has been introduced into mouse *Htt*^38^. We began by confirming that plasma TNFα levels, which we had previously shown to be upregulated at 12 to 14 weeks of age but not at 10 weeks^8^, were increased in our R6/2 colony together with the cytokines that it induces: IL1β, IL6, IL2 and IL10^15,21,39^. The mesoscale discovery (MSD) assay revealed significant increases in TNFα and all of these cytokines in late-stage R6/2 mice, at 14 weeks of age, compared to their wild-type (WT) littermates (Fig. 1). This substantiated the chronic immune activation persistently associated with HD, warranting the assessment of the effects of blocking TNFα activity.

### Intravenous injection with etanercept resulted in the normalization of neurotoxic IL6 in R6/2 plasma

**Figure 2:**
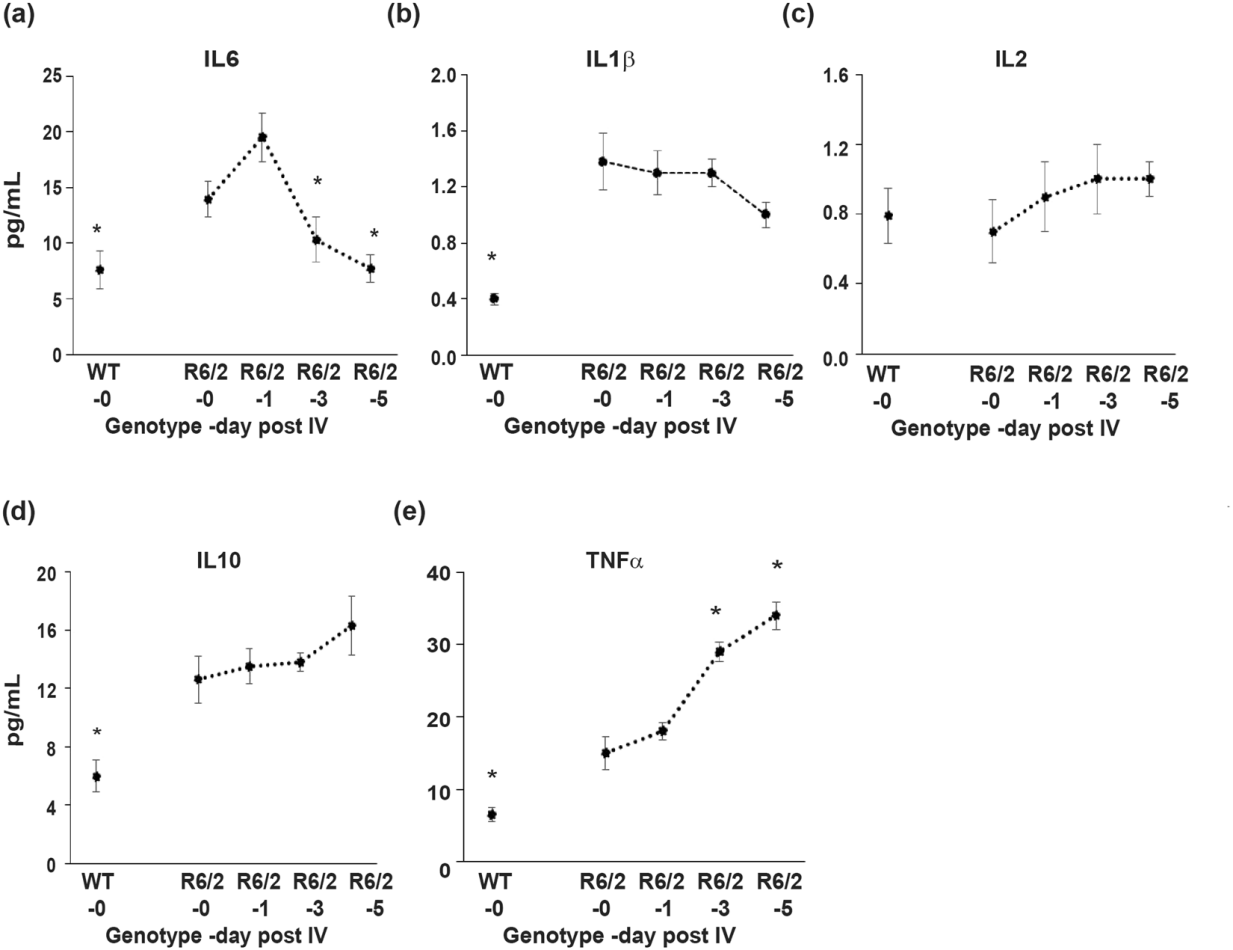
A single IV dose of etanercept effectively reduced IL6 in 13–14 week old R6/2 plasma but significantly increased TNFα levels. Levels of plasma (**a**) IL6, (**b**) IL1β, (**c**) IL2, (**d**) IL10 and (**e**) TNFα following treatment of 13 week old R6/2 mice with a single IV dose of etanercept, as measured by MSD (*n* = 5–9/time point). Cytokine levels were also assessed in WT littermates to determine non-disease associated levels. One-way ANOVA with Bonferroni correction ± SEM. *p < 0.05 vs R6/2 levels at day 0.

In order to determine the effect of TNFα escalation on the cytokine levels of late-stage R6/2 mice, we utilised the TNFα neutralizing drug etanercept, as a means to block TNFα signalling *in vivo*. A single intravenous (IV) dose of 200 μg etanercept decreased plasma IL6 levels by approximately one-third by day 3 post injection, which continued to decrease up until day 5 (Fig. 2a). There was a trend for IL1β levels to progressively decrease for up to 5 days post injection but this failed to reach statistical significance (Fig. 2b). Etanercept treatment had no effect on IL2 (Fig. 2c) or IL10 (Fig. 2d). We were alarmed to see that plasma TNFα levels increased over the 5 day period post injection (Fig. 2e), which might be expected to exacerbate inflammation. However, previous studies have observed similar effects of etanercept treatment on TNFα in patient plasma. This was shown to be the result of a drug “carrier effect”, with etanercept prolonging the blood TNFα half-life, while at the same time blocking its activity^15,40^. We concluded that this might be the basis for the etanercept induced increase in TNFα in R6/2 plasma.

### Etanercept induced reductions in splenic Il6 and Tnfα gene expression in R6/2 mice

**Figure 3:**
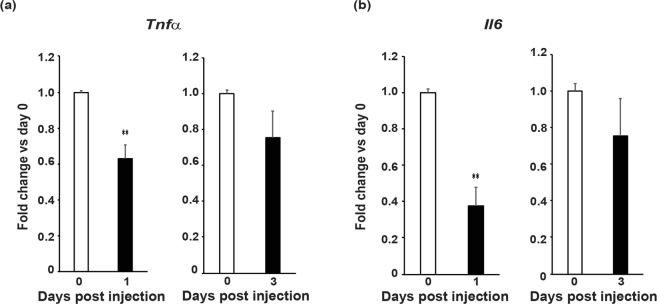
Etanercept treatment reduced splenic *Tnfα* and *Il6* gene expression in R6/2 mice. Gene expression of (**a**) *Tnfα* and (**b**) *Il6* in splenocytes of 13 week old R6/2 mice treated with a single IV dose of etanercept as assessed by real-time qPCR (*n* = 5/group). Student’s *t* test ± SEM. **p ≤ 0.01 vs R6/2 day 0.

To confirm that the increase in TNFα had not occurred due to an increase in production, *Tnfα* gene expression was measured in splenic lymphoid tissue, where cytokine producing immune cells that are activated in HD, reside^8^. Splenic *Tnfα* expression had decreased by approximately 40%, in the etanercept treated mice at day 1 post injection (Fig. 3a). This indicated that the increase in blood TNFα following treatment is unlikely to be due to increased cytokine production in response to drug-induced TNFα signalling inhibition; although, the assessment of other lymphoid tissues is required in order to confirm this definitively. Additionally, since TNFα signalling is able to promote further TNFα production, it is perhaps not unexpected that a significant reduction in splenic *TNFα* gene expression was observed post etanercept treatment^13^. We also measured *Il6* mRNA levels, which were reduced by over 50% one day after IV injection (Fig. 3b), i.e. two days before the plasma IL6 levels decreased (Fig. 2a). Therefore, blocking TNFα activity resulted in decreased *Il6* expression, in the spleen and possibly other lymphoid tissues, as predicted.

### Assessment of the effect of intraperitoneal injection with etanercept on cytokine levels in R6/2 plasma

As it would not be practical to conduct a chronic etanercept study by IV administration, we assessed whether similar reductions in IL6 levels could be achieved through intraperitoneal (IP) administration. Injection of 400 μg of etanercept IP resulted in a trend toward the reduction of IL6 levels after 3 days (*p* = 0.07) (Fig. S1a), IL1β levels were unaffected (Fig. S1b) while increases in both IL2 (Fig. S1c) and IL10 (Fig. S1d) were observed. However, similar to the IV injected mice, TNFα levels were also increased (Fig. S1e). The difference in the effect on IL6 levels may be because insufficient drug was diffusing into blood from the peritoneum, and then subsequently into tissues, with a single 400 μg IP dose of etanercept as compared to IV treatment. We concluded that chronic IP dosing would be necessary to induce a persistent effect on plasma cytokine levels.

### Multiple etanercept IP injections resulted in reductions of IL6 levels after fourteen days of etanercept treatment

**Figure 4:**
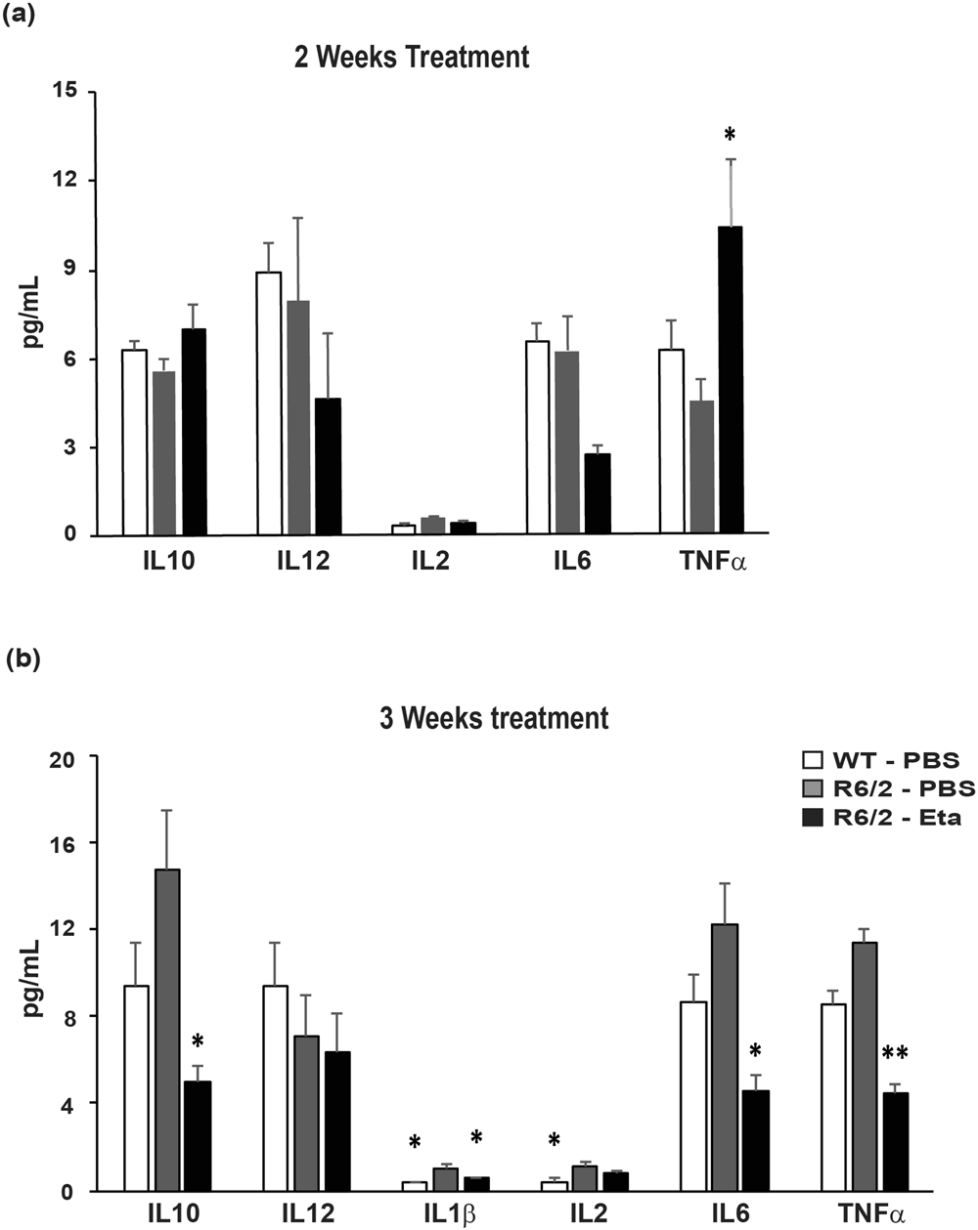
Multiple etanercept IP injections over a two to three week period are required to reduce plasma cytokine levels in R6/2 mice. Plasma IL10, IL12, IL1β, IL2, IL6, and TNFα levels following two (**a**) or three (**b**) weeks treatment of 9–10 week old (at the time of the first injection) R6/2 mice with IP etanercept, or PBS, injections every three days, as measured by MSD (*n* = 5–8/time point). Cytokine levels were also assessed in WT littermates to determine non-disease associated levels. Two-way ANOVA with Bonferroni correction ± SEM. *p < 0.05, **p ≤ 0.01 vs R6/2 PBS treated.

In order to determine whether chronic IP administration of etanercept could induce a persistent decrease in plasma IL6 levels, nine week old R6/2 mice were injected IP with 400 μg of etanercept every 3 days for 3 weeks. After 2 weeks, IL6 levels had decreased by approximately 50% (Fig. 4a). Consistent with the acute IV and IP dosing regimens, TNFα levels had increased (Fig. 4a). After three weeks of etanercept treatment, not only was plasma IL6 reduced (by >50%) but IL1β and IL10 levels were also decreased (by ~55% and ~70% respectively) (Fig. 4b). Futhermore, after three weeks, TNFα was reduced by almost 70% compared to PBS treated mice, probably reflecting a decrease in TNFα autocrine induced production, as well as the degradation of the TNFα that had been stabilized by etanercept binding during the ‘carrier effect’ phase. These data would therefore indicate that the duration of the ‘carrier effect’ in the etanercept treated mice lasts less than three weeks. Since TNFα is able to promote the production of IL10, the observed reduction in this anti-inflammatory cytokine post etanercept treatment was anticipated, however lowering IL10 levels may have detrimental effects on HD mice with already overactive immune responses.

### HD phenotypes were not affected by etanercept treatment of R6/2 mice

Having established a dosing regimen that resulted in the reduction of plasma cytokine levels in R6/2 mice, we went on to assess the effect of chronic etanercept treatment on disease progression. We have previously shown that cytokine induction can be detected in R6/2 macrophages by 8 weeks^8^ and therefore, initiated treatment prior to immune activation. WT and R6/2 female mice were assessed for body weight, grip strength and rotarod performance at 4 weeks of age. Prior to treatment, mice were randomised into their treatment groups according to their body weight, litter of origin and baseline performances for rotarod and grip strength. Mice were treated with etanercept (400 µg) or PBS on Mondays, Wednesdays and Fridays from five weeks of age. One mouse (R6/2-PBS treatment group) died at nine weeks of age and was removed from the study. Efficacy was investigated by the longitudinal assessment of body weight, grip strength, rotarod performance and exploratory activity in the open field, and the investigator was blind to treatment status.

**Figure 5:**
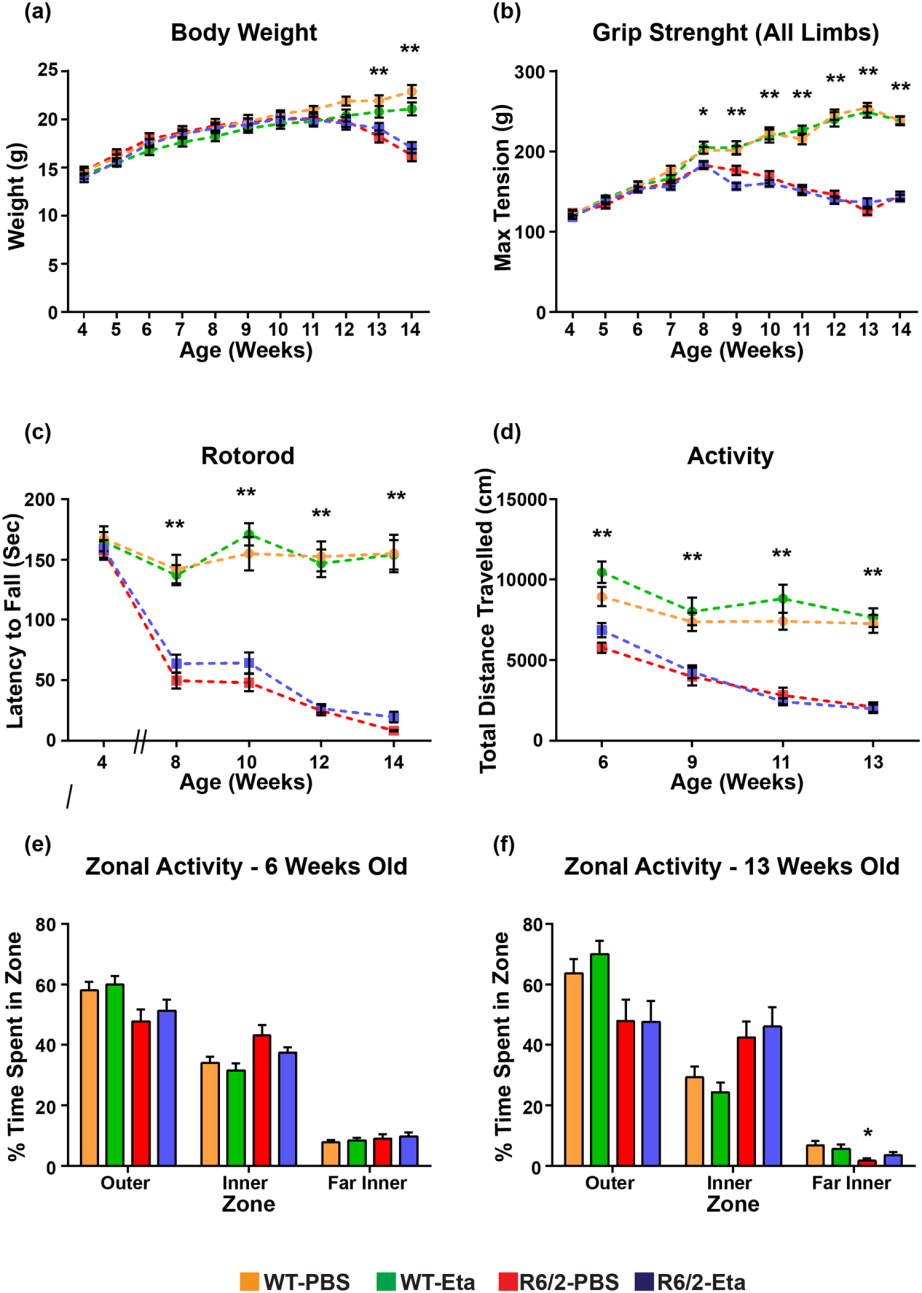
Treatment with etanercept had no effect on phenotype progression in female R6/2 mice. Longitudinal phenotypes (**a**) body weight (**b**) grip strength (all limbs), (**c**) rotarod performance (**d**) exploratory activity (30 min) (**e**,**f**) thigmotaxis were measured from 4 to 14 weeks of age as indicated (WT-PBS *n* = 16; R6/2-PBS *n* = 13; WT-Eta *n* = 15; R6/2-Eta *n* = 16). GLM/repeated measures ANOVA ± SEM *p *≤* 0.05, **p *≤* 0.01 (WT-PBS vs R6/2-PBS).

Body weight was measured weekly from four to fourteen weeks of age on Mondays. Consistent with previous data, R6/2 mice failed to continue to gain weight relative to WT littermates [F(Age × Genotype)_10,550_ = 46.326, *p* < 0.001] (Fig. 5a). This failure to gain weight was not alleviated by etanercept [F(Age × Genotype × Treatment)_10,550_ = 0.963, *p* = 0.475], indicating that etanercept treatment had no beneficial effects on R6/2 weight loss.

Grip strength for all four limbs was measured at the beginning of each week from four to fourteen weeks of age. R6/2 mice had impaired grip strength from eight weeks of age relative to WT littermates [F(Age × Genotype)_10,550_ = 91.771, *p* < 0.001] (Fig. 5b) which was not ameliorated upon etanercept treatment [F(Age × Genotype × Treatment)_10,550_ = 0.783, *p* = 0.602].

Mice were tested on an accelerating rotarod at four, eight, ten, twelve and fourteen weeks of age. There was no difference between R6/2 and WT littermates in motor performance at four weeks of age [F(Genotype)_3,56_ = 4.151, *p* = 0.091] (Fig. 5c), with a decline of performance initiating from eight weeks of age [F(Age × Genotype)_5,250_ = 43.011, *p* < 0.001]. Etanercept failed to rescue rotarod impairment over the course of the trial [F(Age × Genotype × Treatment)_5,250_ = 1.624, *p* = 0.171].

Exploratory activity in the open field was measured at six, nine, eleven and thirteen weeks of age. An overall change in activity, as general exploration/total distance moved (in an open field arena) was observed at the earliest timepoint measured (six weeks of age) between R6/2 and WT littermates [F(Genotype)_(3,54)_ = 14.526, *p* < 0.001] (Fig. 5d) independent of treatment received. This genotype difference was observed at all other time point measured and was exacerbated with age [F(Age × Genotype)_3,117_ = 10.304, p < 0.001] but not rescued by etanercept treatment [F(Age × Genotype × Treatment)_3,117_ = 0.470, *p* = 0.677]. Diminished exploratory behaviour was not accompanied by anxiety in these animals. R6/2 did not exhibit changes in thigmotaxis compared to WT over the course of the trial [F(Age × Genotype)_3,129_ = 1.968, *p* = 0.135] (Fig. 5e,f).

### Etanercept partially attenuated the decrease in brain weight that occurs in R6/2 mice

**Figure 6:**
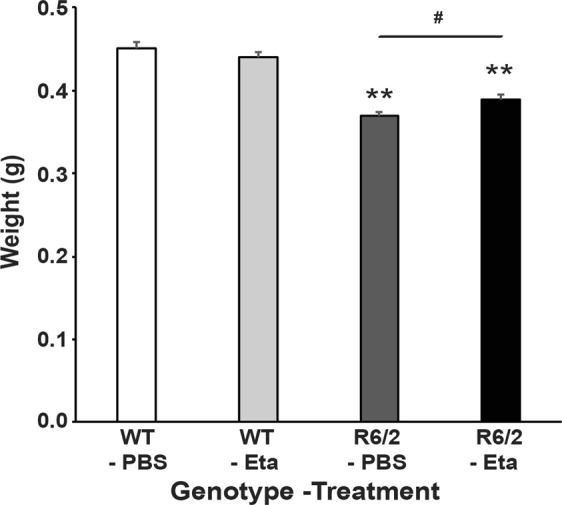
Etanercept treatment partially attenuated the decrease in brain weight that occurs in R6/2 mice. Fresh brains were weighed at the end of the study (at 14 weeks of age) (*n* = 12–15/genotype-treatment group). Two way ANOVA with Bonferroni correction ± SEM **p < 0.01 (vs WT-PBS), ^**#**^*p* < 0.02 (R6/2-PBS vs R6/2-Eta).

A progressive decrease in brain weight occurs in R6/2 mice^41^ and therefore, we measured the fresh brain weights at study termination (14 weeks of age). The brains of R6/2 mice treated with either PBS or etanercept weighed less than those of the WT-PBS treatment group (Fig. 6). However, the brains of R6/2 mice treated with etanercept were heavier than those treated with PBS by approximately 25 g (Fig. 6).

### Etanercept failed to impact on striatal TNFα and IL6 levels in both WT and R6/2 mice

**Figure 7:**
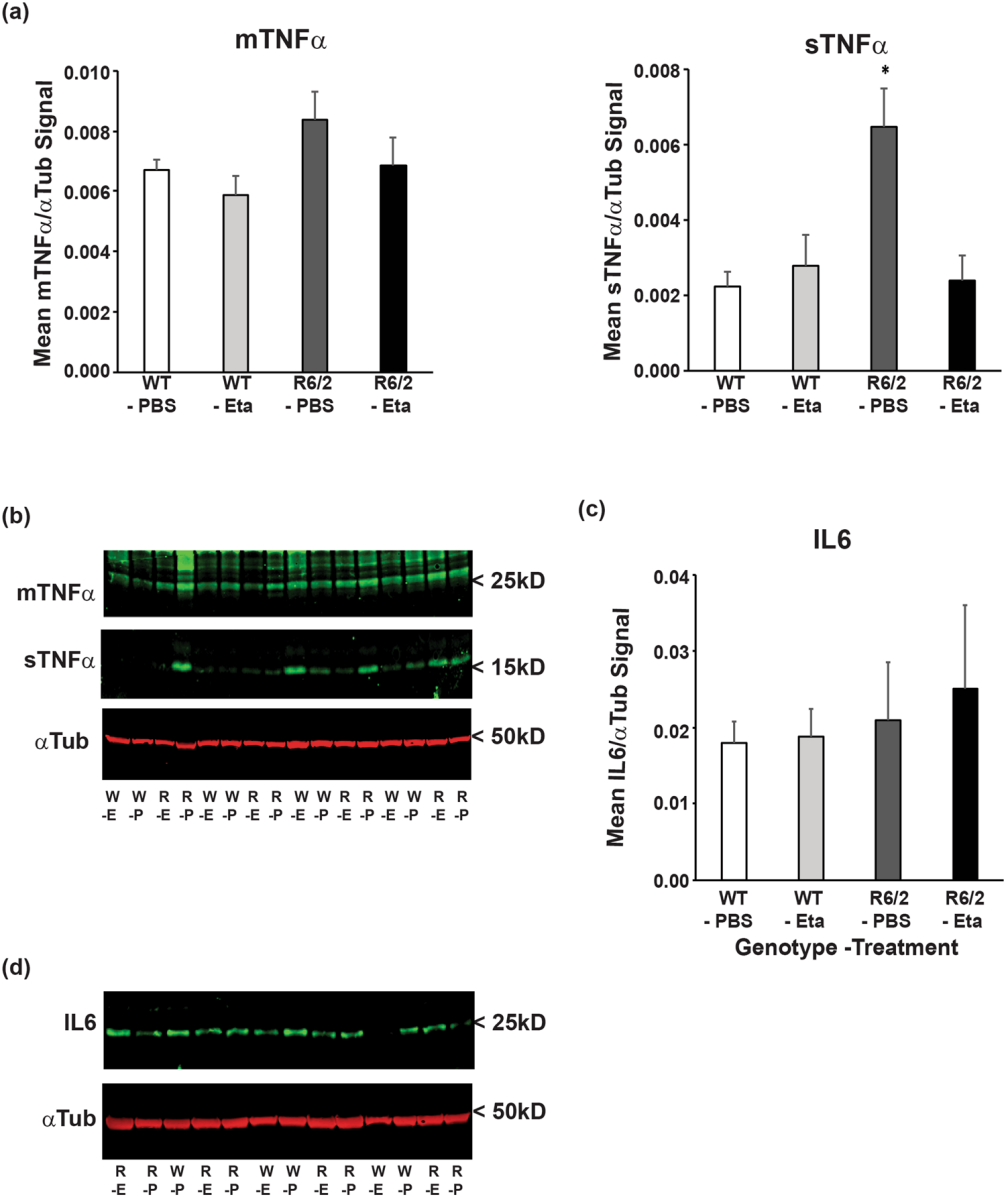
Etanercept treatment lowered the levels of soluble TNFα in the R6/2 striatum. Striatal tissue lysates of 14 week old mice, obtained three days after the final injection were immunoprobed for their TNFα and IL6 content by western blot analysis. (**a**) mTNFα and sTNFα levels normalised to αTub ± SEM. (**b**) A representative western blot for mTNFα, sTNFα and αTub. (**c**) IL6 normalised to αTub ± SEM (**d**) a representative western blot for IL6 and αTub. *N* = 11–12/genotype-treatment group). *p < 0.05 (vs WT-PBS), by two way ANOVA with Bonferroni correction. mTNFα = membrane-bound TNFα, sTNFα = soluble TNFα, αTub = α-tubulin, R = R6/2, W = WT, −P = PBS treated, −E = etanercept treated. Complete western blots are shown in Figs S2 and S3.

Cortical and striatal TNFα levels are increased in late stage R6/2 mice^8,36^ and therefore we investigated whether treatment with etanercept had altered striatal TNFα levels. Western blot analysis of membrane bound (mTNFα) and soluble (sTNFα) (Fig. 7a,b) showed that sTNFα was increased in R6/2-PBS striatum as compared to WT-PBS by approximately three fold, as previously reported^36^. However, the levels of both TNFα forms were low and, as measured by this approach, were quite variable between mice. Although there was no difference in the levels of sTNFα between WT-PBS and R6/2-Eta, the decrease in R6/2 striatal sTNFα levels with treatment were not statistically significant (*p* = 0.215).

In contrast to *Tnfα*, we did not previously observe any increases in *Il6* gene expression in late stage R6/2 mice striatum^8^. However since IL6 levels are increased in the plasma of these mice (Fig. 1), and IL6 is capable of crossing the BBB^42^, this cytokine could have migrated from plasma to the brain, resulting in an increase in brain levels. Therefore, we measured striatal IL6 levels by western blot, but found no evidence for increased IL6 in late stage R6/2 striatum and no effect of etanercept (Fig. 7c,d), despite the reduction in plasma IL6 levels in response to treatment (Fig. 2a).

## Discussion

Immune system hyper-activation and chronic inflammation has been consistently documented and is recognized as a feature of HD. We and others have observed inflammatory cytokine increases, along with heightened immune cell activation, not only in innate immune cells^1,8,27,42^ but also possibly within adaptive T lymphocytes^8^. Previous studies have reported that immunomodulation in HD mouse models has therapeutic benefits^35,36^, thus immunosuppression may provide a potential therapeutic strategy for HD. In view of this, we investigated whether TNFα might contribute to disease pathology, not only because of its’ possible neurotoxic effects coupled with its ability to cross the BBB barrier, but also because of its’ capacity to upregulate other neurotoxic cytokines, such as IL1β and IL6, which are also dysregulated during HD^1,8,29^. We postulated that TNFα plays a central role in promoting or driving the inflammatory milieu observed in HD mice and patients alike, and hypothesised that targeting this specific immune factor alone may impact on HD-associated inflammation, and potentially disease progression. Targeting TNFα as a potential HD treatment received further credence from the accessibility of the TNFα signalling inhibitor, etanercept, which is currently the treatment of choice for a number of autoimmune diseases.

Herein, we show that *in vivo* inhibition of TNFα activity via etanercept was able to normalise neurotoxic IL1β, IL6 as well as TNFα itself in R6/2 plasma during chronic treatment. However, a longitudinal assessment of disease-associated phenotypes from five weeks of age revealed that etanercept therapy had no effect on the extent or progression of weight loss, grip strength impairment, exploratory hypoactivity or rotarod performance. Elevated IL10 levels observed in R6/2 mice were also unsurprisingly reduced following etanercept treatment, and since this cytokine has anti-inflammatory activity, this could have deleterious consequences in this setting of chronic inflammation. However, our nine-week treatment study did not detect any such undesirable effects.

Interestingly, we found that the reduction of brain weight in 14 week old R6/2 mice had been partially attenuated by etanercept treatment. We had previously found that *Tnfα* levels were increased in the striata of R6/2 mice as compared to WT, but not in the cortex and cerebellum^8^. This prompted us to measure TNFα levels within the striata of mice from the four treatment groups. If these levels were modulated by etanercept, which does not cross the BBB^43^, it would suggest that peripheral sTNFα was contributing to the striatal levels either directly, by supplementing the existing sTNFα concentration in the striatum and/or indirectly, by stimulating local striatal sTNFα production. We found that sTNFα levels were increased in R6/2 mice and that there were signs that these were normalised by etanercept. This etanercept effect might have been more pronounced had samples been taken earlier, rather than three days after the last dose, as by this time some recovery in TNFα levels may have occurred (Fig. 3a). Our data suggest that sTNFα originating in the periphery could contribute to striatal levels and that peripheral drug administration may therefore act centrally within the brain through its effects on peripheral sTNFα production.

Intriguingly, one of the major downstream targets of TNFα signalling is the NFκB transcription factor^44^ which is involved in promoting cytokine production when activated^45^. Furthermore, studies have shown that NFκB activation is amplified in HD^29,46^ which prompted us to assess whether etanercept exerted its effects via NFκB. We attempted to analyse NFκB activation via quantification of phosphorylated (p)IκB^47^ protein levels in spleen, but did not detect any differences. Although the result could be interpreted as indicating that NFκB responses are not dysregulated in the lymphoid tissue of R6/2 mice, a more plausible explanation might be that we did not investigate the relevant immune cell populations in our assay. Since the spleen is composed of a large proportion of non-immune cells, as well as immune cell populations that may not be affected in HD, these may have masked any differences in NFκB activity in the R6/2 macrophage cell population. Follow up studies on NFκB activity post etanercept treatment, specifically within purified cells that are known to be affected in R6/2 mice such as macrophages and dendritic cells^8,48^ and are therefore needed.

The beneficial effects of treating R6/2 mice with ZPro1595, a dominant negative inhibitor of sTNFα, that was diffused directly into the brain, have previously been reported^36^. We did not find the peripheral administration of etanercept to be efficacious, which indicates that the route of administration may impact drug efficacy. Our preclinical mouse data do not support etanercept treatment, via the peripheral route of delivery, as a therapy for HD.

## Materials and Methods

### Mouse maintenance and breeding

All animal care and procedures were performed in compliance with United Kingdom Home Office regulations (Animals and Scientific Procedures Act 1986) and were approved by the University College London Ethical Review Process Committee. Hemizygous R6/2 mice were bred by backcrossing R6/2 males to (CBA/Ca × C57BL/6J) F1 females (B6/CBAF1/OlaHsd Envigo, UK). R6/2 mice are transgenic for the 5′ region of the mutant human huntingtin (*HTT*) gene^37^ which expresses an exon 1 HTT protein. Same gender mice were group-housed with mixed genotypes in each cage. Animals were kept on a 12 hour light and dark cycle and room temperature was maintained at 21 °C ± 1 °C. All mice had access to moderate environmental enrichment (play tube, wooden chew sticks). Cages were provided with an unlimited supply of water and a high protein chow diet. From 12 weeks of age, a mashed diet was additionally provided. Mice were genotyped and the *HTT* CAG repeat length was quantified as previously described^49^. The mean CAG repeat was 185.80 ± 6.88 (SD). Mice were monitored weekly using a humane endpoint scale as previously published^50^ and euthanized if endpoint had been reached.

### Blood/plasma and tissue sample collection

Blood was taken via tail vein puncture then by decapitation post cervical dislocation and collected into EDTA tubes. Blood samples were spun at 2000 × *g* for 5 min and the upper plasma layer removed. Following euthanasia, whole spleens tissues were dissected and single cell suspensions were obtained as described previously^51^. All splenocyte samples were treated with red blood cell lysis solution (R&D Systems) in order to remove red blood cells prior to processing.

### Plasma analyses

IL1β, IL2, IL6, IL10, IL12 and/or TNFα levels in plasma were quantified using Meso Scale Discovery (MSD) assays in accordance with the manufacturer’s instructions and analysed on a SECTOR 2400 instrument (MSD).

### Assessment of etanercept treatment on cytokine levels after acute and chronic dosing

Thirteen to fourteen week old female mice were injected intravenously (IV) with 200 µg etanercept via the tail vein or with 400 µg etanercept intraperitoneally (IP). Plasma was collected for cytokine measurement by MSD at day 0 and at specified days post treatment, and levels were compared to that in plasma from untreated WT mice. Spleens were also collected from the IV injected mice for qPCR analysis. A chromic IP dosing study was also performed in which R6/2 mice were injected every 3 days with 400 µg etanercept or PBS for either 2 or 3 weeks. Plasma cytokine levels were compared to those in WT mice treated with PBS.

### RNA extraction and quantitative real time gene expression analysis

Splenocytes were isolated as described previously^51^. Total RNA from splenocytes was extracted with the mini-RNA kit according to the manufacturer’s instruction (Qiagen). Using the MMLV Superscript reverse transcriptase (Invitrogen) and random hexamers (Operon), reverse transcription (RT) was performed as described previously^52^. Taqman quantitative real-time PCR (qPCR) was performed on the Chromo4 real-time PCR detector (Biorad) as described previously and quantified via the 2^−ΔΔCT^ method^52,53^. The expression levels of a ‘gene of interest’ was normalised to the geometric mean of endogenous housekeeping genes (*B2m* and *Atp5b*). Primer and probe sets for *Il6 and Tnfα*, as well as *B2m* and *Atp5b* housekeeping genes were from Life Technologies, while real time PCR mastermix (SsoAdavanced Universal Probes Supermix) were purchased from BioRad.

### Protein extraction, SDS PAGE and immunoblotting

Frozen mouse left and right striatum was homogenized in RIPA lysis buffer (Abcam) containing complete protease inhibitors (Roche) with a polytron homogenizing probe. Samples were sonicated at 4 °C using a vibracell sonicator (10 × 1 s 20 kHz pulses) and spun at 13,000 × *g* for 20 min at 4 °C. The protein concentration of the supernatant was determined by the BCA assay (Thermo Scientific) according to the manufacturer’s protocol. Protein lysates were diluted with 4x NuPage LDS buffer (Life Technologies), denatured for 10 min at 95 °C, loaded onto 10% SDS polyacrylamide gels and subjected to western blotting onto nitrocellulose membranes using the Trans-Blot Turbo RTA Transfer Kit and Trans-Blot Turbo Transfer System (Bio-Rad) according to the manufacturer’s instructions. Membranes were blocked in Odyssey Blocking Buffer-TBS (Li-Cor) containing 0.2% Tween 20 (BB-T) overnight at 4 °C. Primary antibodies against mouse IL6 (Stratech Scientific, A00102-2-WBO) or TNFα (Bio Techne, AF-410-NA) and α-tubulin (αTub) (Sigma, MAB1637) were incubated overnight at 4 °C in BB-T. Blots were washed three times for 5 min in Tris buffered saline (TBS) containing 0.1% Tween 20 (TBS-T) and incubated for 1 hr at RT with the appropriate secondary antibodies conjugated with IRDye 800CW or IRDye 680RD (Li-Cor) in BB-T. Blots were washed three times for 5 min in TBS-T, and the target protein band fluorescence signal quantified using the Odyssey Image Studio System (Li-Cor). The fluorescence signal of an appropriate area devoid of bands on the negative (lysis buffer) control lane was subtracted from the values obtained for bands of interest in order to normalize against background. Relative fluorescence levels were determined by dividing the normalized fluorescence readings of the bands of interest by the corresponding αTub loading control band of each sample.

### Etanercept efficacy trial

WT and R6/2 female mice were treated with 400 μg of etanercept in 400 μl PBS, or with 400 μl PBS as a vehicle control, from five weeks of age on Mondays, Wednesdays and Fridays. Prior to treatment, WT and R6/2 mice were assessed for their baseline grip strength and rotarod performance at four weeks of age and then randomised into their treatment groups according to their body weight, baseline performance and litter of origin (WT-PBS, n = 16; R6/2-PBS n = 13; WT-Eta n = 15; R6/2-Eta n = 16). A reduction in body temperature can be indicative of an adverse response to a treatment. Body temperature was monitored weekly from five to ten weeks of age, using an infra-red temperature reader (ThermoScan Instant Thermometer, Braun) that was reproducibly positioned under the thorax. There was no change in body temperature, which, taken together with other measures of appearance, indicated that the treatment was well- tolerated.

Efficacy was investigated by measuring body weight, grip strength, rotarod performance and exploratory activity in the open field and the investigator was blind to treatment status. Body weight was measured weekly from four to fourteen weeks of age at the beginning of the week.

Grip strength for all four limbs was measured weekly from four to fourteen weeks of age as previously described^54,55^ (Bioseb, *In Vivo* Research Instruments, and USA). At the beginning of each week, the maximum tension (g) was measured for five trials and the average of the highest three grip strength readings was used.

Mice were tested on an accelerating rotarod (Ugo Basile 7650, Italy) as previously described^54,56^ at four, eight, ten, twelve and fourteen weeks of age. For each trial, mice were allowed to acclimatise to the rotating drum for 20 sec before it began to accelerate from 4–40 rpm for a maximum of 300 sec. The rotating drum was adapted with smooth bicycle inner tubing to prevent mice from gripping onto the rod. Latency to fall from the rotating rod (sec) was measured as a readout for motor function. Mice were tested for three trials per day for four days at four weeks of age and then for three trials per day for three days per week for the remainder of the experiment. Mice were rested for a minimum of 1 hour between each trial. Rotarod paddles and drums were thoroughly cleaned with 70% industrial methylated spirit (IMS) between each mouse. The mean latency to fall times for each mouse group at specified time points were calculated as described previously^54^.

Exploratory activity in the open field was measured at six, nine, eleven and thirteen weeks of age, as previously described^55^. Mice were individually placed in a custom built 50 cm diameter square, white open field arena (Engineering & Design Plastics Ltd., Cambridge, UK) for 30 min in order to assess exploratory behaviour in a novel environment. The total distance travelled (cm) and mean velocity (cm/s) of each mouse was recorded by video camera (Euresys Picolo Diligent Plus, RMA electronics, MA USA) and analysed by a video software tracking centre-point detection (Ethovision XT, Noldus, Netherlands). The open field arena was divided into three zones by squares drawn at equi-distances from the outer walls (created using the EthovisionXT v11.5 software), thus creating a far inner-, inner- and outer-zone. Thigmotaxis, the time spent in the peripheral outer-zone of an open field, is indicative of an anxiety-like behaviour. Measures of total time spent in each zone were analysed. The open field arena was cleaned using 70% IMS between each set of mice.

### Statistical analysis

Differences between specified groups were detected using Student’s *t*-test (Microsoft Excel) or ANOVA with Bonferroni correction where appropriate (IBM SPSS Statistics Ver.22), as stated in the Figure legends. All data were screened for statistical outliers using ROUT Test (GraphPad Software) and outlier values were excluded from the analysis. A GLM/repeated measures ANOVA was used with genotype, age and treatment as within- subject factors (Greenhouse–Geisser correction for non sphericity) for all longitudinal tests to determine the effect of either genotype or treatment over the course of the trial. Data were plotted using Prism Ver.6.0 (GraphPad Software). *P-values* of <0.05 were considered significant.

## Supplementary information

Supplementary information

## Data Availability

The datasets generated during and/or analysed during the current study are available from the corresponding author on reasonable request.
