# Supplementary material for: Inhibition of tumour necrosis factor alpha in the R6/2 mouse model of Huntington’s disease by etanercept treatment
Source: Sci Rep. 2019 May 10;9:7202. doi: 10.1038/s41598-019-43627-3 (PMC6510744; doi:10.1038/s41598-019-43627-3)
Supplement: Supplementary file 1 — Supplementary information [file 41598_2019_43627_MOESM1_ESM.pdf]

**Supplementary Information**

# Inhibition of tumour necrosis factor alpha in the R6/2 mouse model of Huntington's disease by etanercept treatment.

Jeffrey Pido-Lopez[1], Benedict Tanudjojo[1,2], Sahar Farag[1,], Marie Bondulich[1], Ralph Andre[1], Sarah J. Tabrizi[1] and Gillian P. Bates[1].

1. Huntington's Disease Centre, Department of Neurodegenerative Disease and Dementia Research Institute, UCL Queen Square Institute of Neurology, University College London, London WC1N 3BG, UK.

2. Nuffield Department of Clinical Neurosciences, John Radcliffe Hospital, University of Oxford, Oxford OX3 9DU.

Corresponding author: j.pido@ucl.ac.uk or gillian.bates@ucl.ac.uk

## Supplementary Figures

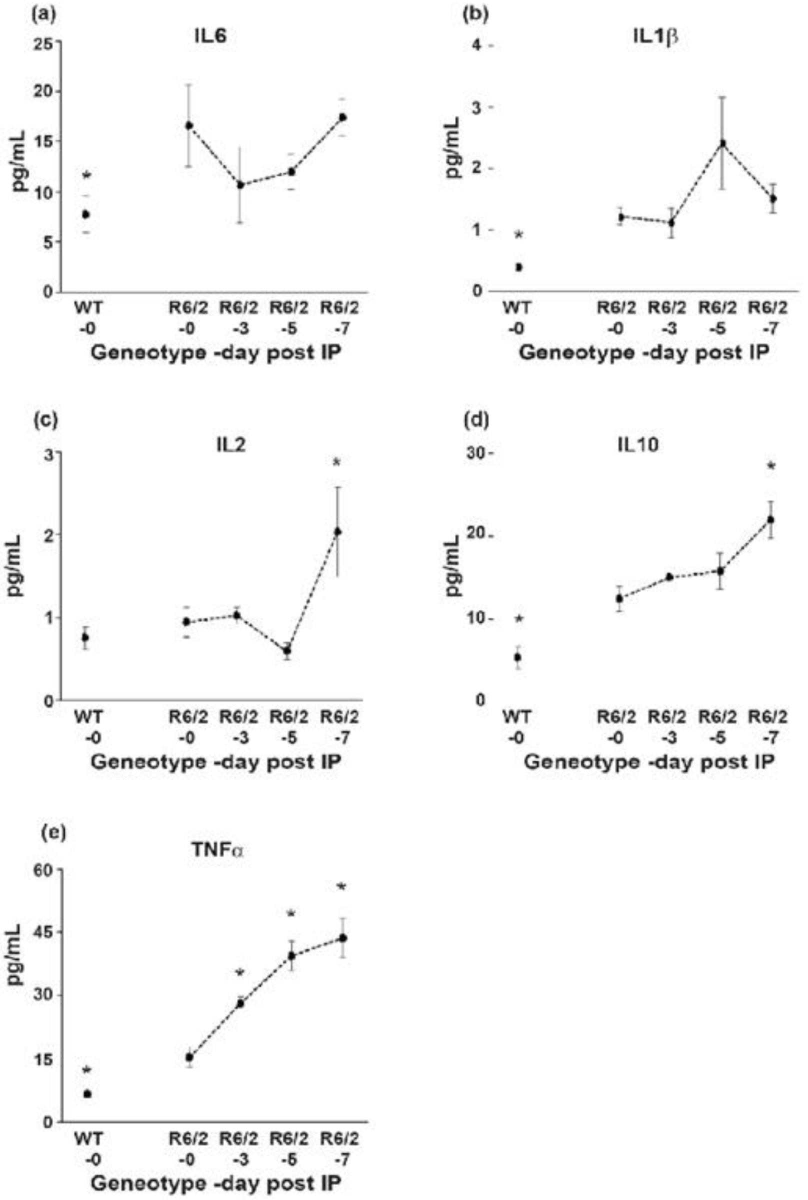


**Supplementary Figure 1. A single IP dose of etanercept did not reduce IL6 levels in late stage-R6/2 plasma but still significantly increased TNFα.** Levels of plasma (**a**) IL6, (**b**) IL1β, (**c**) IL2, (**d**) IL10 and (**d**) TNFα following treatment of 13 week old R6/2 mice with a single IP etanercept injection, as measured by MSD (*n*= 5-8/time point). Cytokine levels were also assessed in WT littermates to determine non-disease associated levels. One-way ANOVA with Bonferroni correction ± SEM. ∗ *p* <0.05 vs R6/2 at day 0.

a)

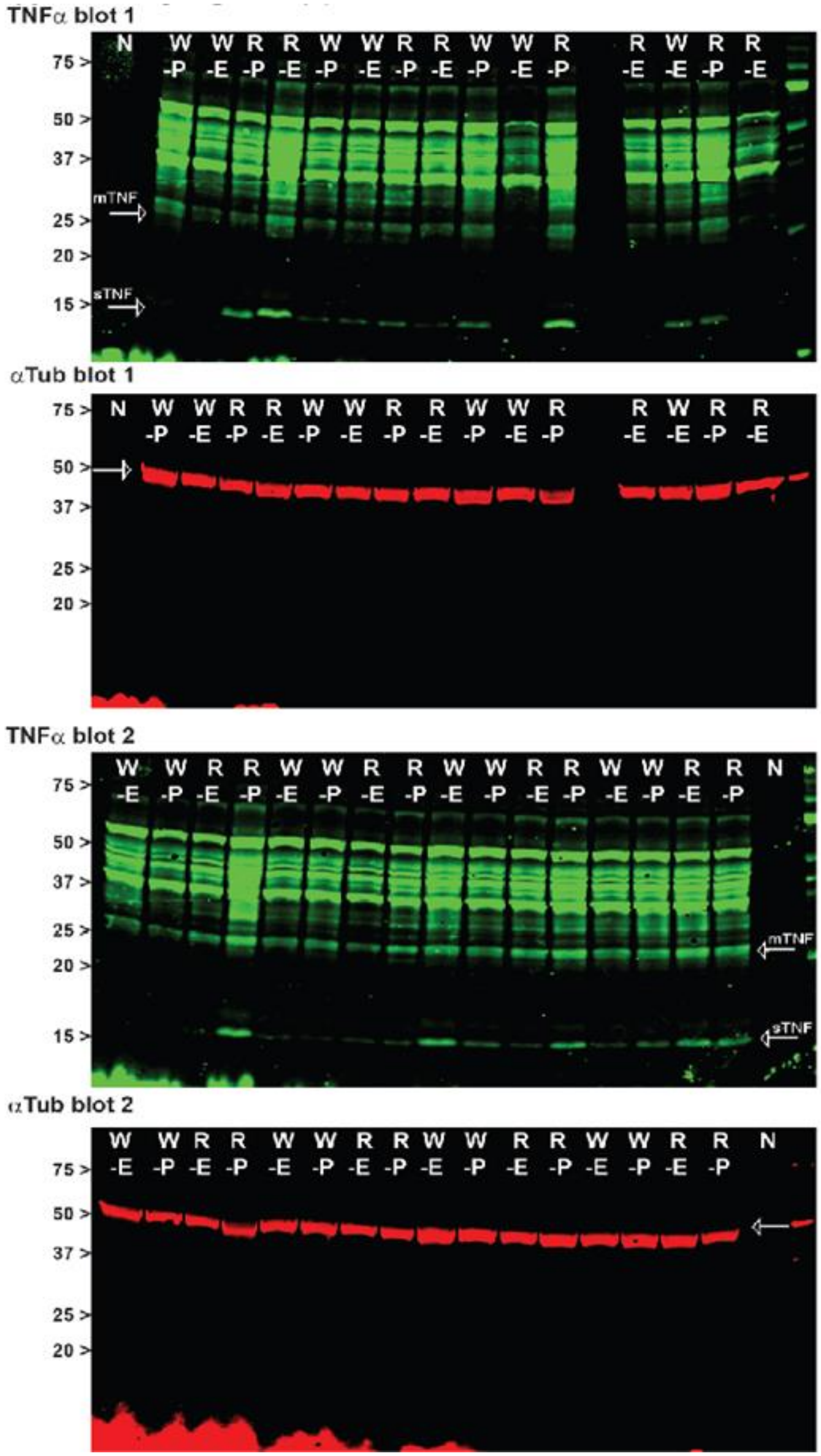
TNFα blot 1
N W W R R W W R R W W R R W R R
-P -E -P -E -P -E -P -E -P -E -P -E -E -P -E
75 >
50 >
37 >
mTNF
25 >
20 >
sTNF
15 >
αTub blot 1
N W W R R W W R R W W R R W R R
-P -E -P -E -P -E -P -E -P -E -P -E -E -P -E
75 >
50 >
37 >
25 >
20 >
TNFα blot 2
W W R R W W R R W W R R W W R R N
-E -P -E -P -E -P -E -P -E -P -E -P -E -P -E -P
75 >
50 >
37 >
25 >
mTNF
20 >
15 >
sTNF
αTub blot 2
W W R R W W R R W W R R W W R R N
-E -P -E -P -E -P -E -P -E -P -E -P -E -P -E -P
75 >
50 >
37 >
25 >
20 >

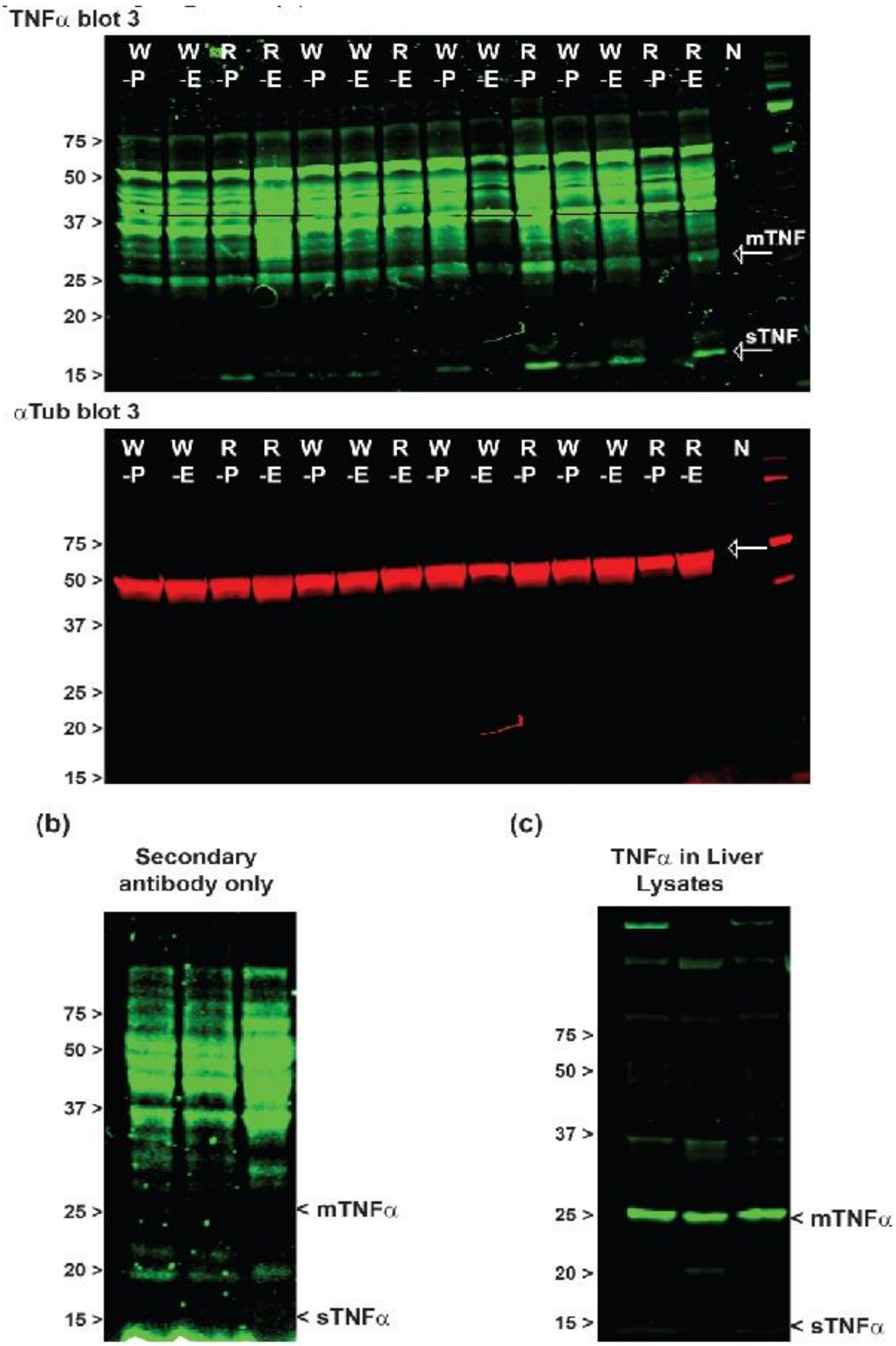

TNFα blot 3
W -P
W -E
R -P
R -E
W -P
W -E
R -E
W -P
W -E
R -P
W -P
W -E
R -P
R -E
N
75 >
50 >
37 >
25 >
20 >
15 >
mTNF
sTNF
αTub blot 3
W -P
W -E
R -P
R -E
W -P
W -E
R -E
W -P
W -E
R -P
W -P
W -E
R -P
R -E
N
75 >
50 >
37 >
25 >
20 >
15 >
(b)
Secondary antibody only
75 >
50 >
37 >
25 >
20 >
15 >
< mTNFα
< sTNFα
(c)
TNFα in Liver Lysates
75 >
50 >
37 >
25 >
20 >
15 >
< mTNFα
< sTNFα

**Supplementary Figure 2. Western blot images for TNFα in striatal lysates of 14 week old WT and R6/2 mice treated with PBS or etanercept over nine weeks.** TNFα blots and their corresponding α-tubulin (αTub) blots (x 3 blots) are shown with white arrows indicating the position of the target protein bands of ~16 kD for sTNFα (soluble), ~26 kD for mTNFα (membrane-bound) and ~50 kD for αTub **(a)**. The secondary antibody cross reacted with several proteins in striatal lysates, however no cross reacting bands were observed at the ~16 kD or 26 kD levels indicating that the bands migrating at these sizes represent the sTNFα and mTNFα proteins respectively **(b and c)**. The secondary antibody cross reacts with proteins in striatal lysates **(b)** but less in liver lysates **(c)**, where only a few faint non-specific bands can be detected; we did not detect sTNFα in liver lysates. R = R6/2, W = WT, –P = PBS treated, -E = etanercept treated, N = negative control.

In order to prevent the cross reacting striatal proteins competing for the secondary antibody with TNFα, the nitrocellulose membranes were cut horizontally into two parts at approximately the 35 kD level and each part stained separately with the secondary antibody. The two parts of the membrane were washed separately and subsequently realigned during the visual analysis and signal quantification of the membrane.

IL6 blot 1

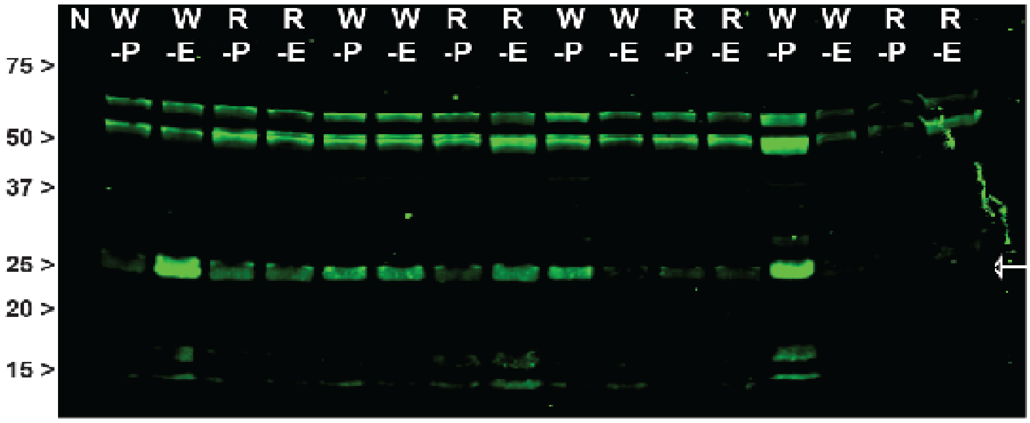


αTub blot 1

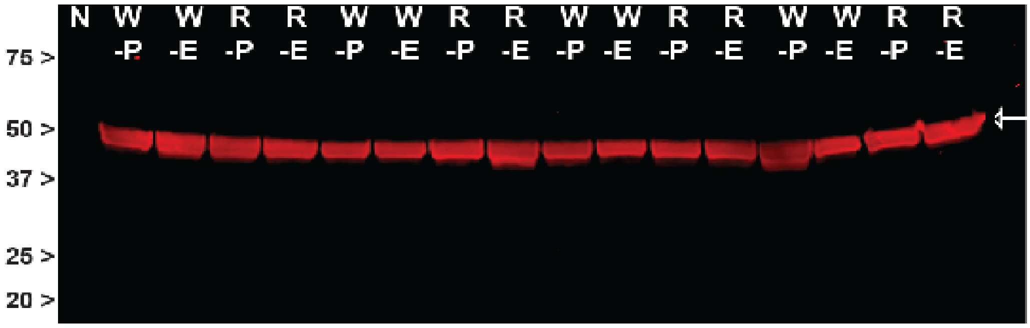


IL6 blot 2

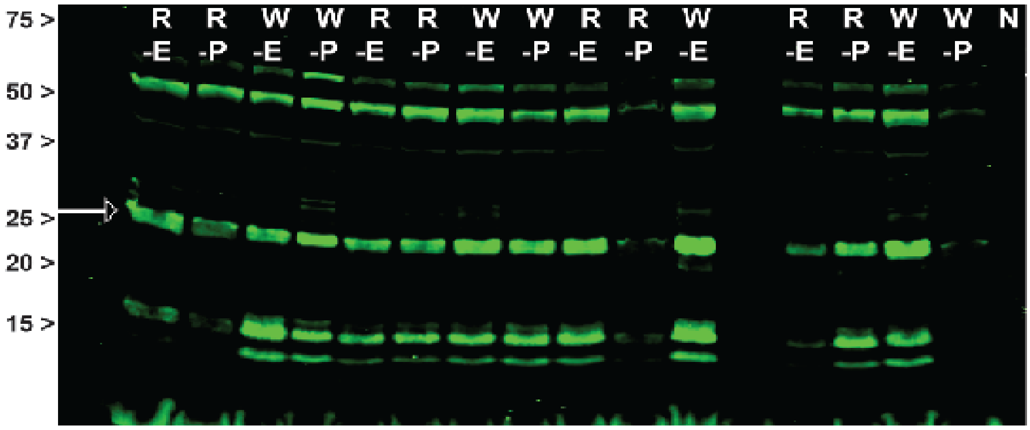


αTub blot 2

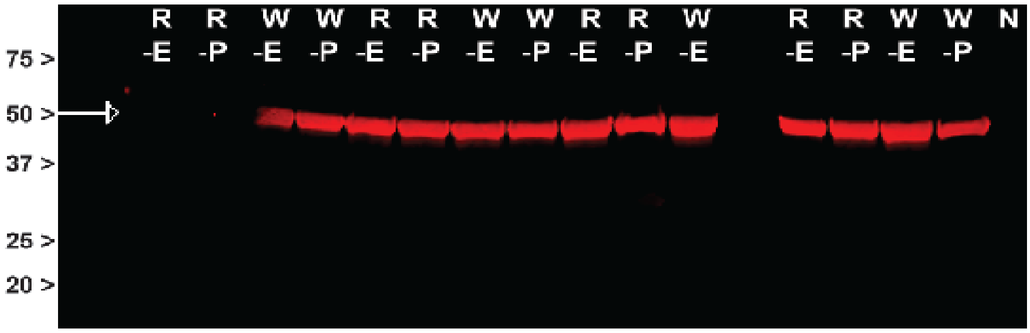

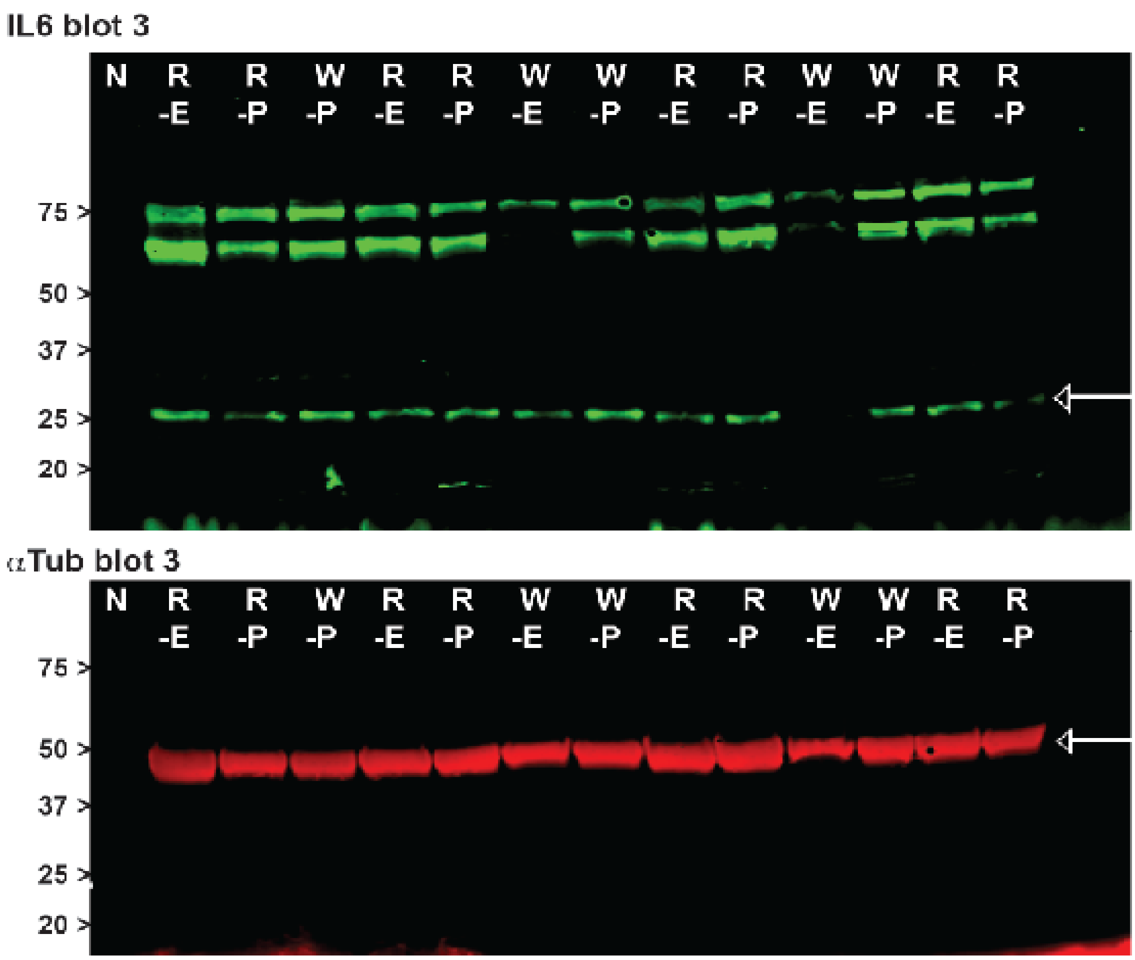


**Supplementary Figure 3. Western blot images for IL6 in striatal lysates of 14 week old WT and R6/2 mice treated with PBS or etanercept over nine weeks.** IL6 blots and their corresponding α–tubulin (α Tub) blots (x 3 blots) are shown with white arrows indicating the position of the target protein bands of ~25 kD for IL6 and ~50 kD for αTub. Additional bands at ~55 and ~65 kD on the IL6 blots may represent glycosylated forms of IL6. R = R6/2, W = WT, –P = PBS treated, -E = etanercept treated, N = negative control.
